# A Review of Automation and Sensors: Parameter Control of Thermal Treatments for Electrical Power Generation

**DOI:** 10.3390/s24030967

**Published:** 2024-02-01

**Authors:** William Gouvêa Buratto, Rafael Ninno Muniz, Ademir Nied, Carlos Frederico de Oliveira Barros, Rodolfo Cardoso, Gabriel Villarrubia Gonzalez

**Affiliations:** 1 Electrical Engineering Graduate Program, Department of Electrical Engineering, Santa Catarina State University (UDESC), Joinville 89219-710, Brazil; 2 Electrical Engineering Graduate Program, Department of Electrical Engineering, Federal University of Pará (UFPA), Belém 66075-110, Brazil; 3 Production Engineering Graduate Program, Department of Science and Technology, Federal Fluminense University (UFF), Rio das Ostras 28895-532, Brazil; 4 Expert Systems and Applications Lab, Faculty of Science, University of Salamanca, 37008 Salamanca, Spain

**Keywords:** automation and sensors, data acquisition, parameter control, thermal treatments, electrical power generation

## Abstract

This review delves into the critical role of automation and sensor technologies in optimizing parameters for thermal treatments within electrical power generation. The demand for efficient and sustainable power generation has led to a significant reliance on thermal treatments in power plants. However, ensuring precise control over these treatments remains challenging, necessitating the integration of advanced automation and sensor systems. This paper evaluates the pivotal aspects of automation, emphasizing its capacity to streamline operations, enhance safety, and optimize energy efficiency in thermal treatment processes. Additionally, it highlights the indispensable role of sensors in monitoring and regulating crucial parameters, such as temperature, pressure, and flow rates. These sensors enable real-time data acquisition, facilitating immediate adjustments to maintain optimal operating conditions and prevent system failures. It explores the recent technological advancements, including machine learning algorithms and IoT integration, which have revolutionized automation and sensor capabilities in thermal treatment control. Incorporating these innovations has significantly improved the precision and adaptability of control systems, resulting in heightened performance and reduced environmental impact. This review underscores the imperative nature of automation and sensor technologies in thermal treatments for electrical power generation, emphasizing their pivotal role in enhancing operational efficiency, ensuring reliability, and advancing sustainability in power generation processes.

## 1. Introduction

Automation and sensor technologies play a crucial role in optimizing and enhancing various aspects of thermal treatments across different industries. When combined, they allow precise control over temperature, pressure, and other critical parameters during thermal treatments. This contributes to overall energy efficiency and sustainability in thermal treatment operations [1]. These technologies are integral components of the Industry 4.0 paradigm, facilitating the integration of digital equipment for a connected and intelligent manufacturing environment [2]. This includes the use of the Internet of Things (IoT) [3], programmable logic controllers (PLCs) [4], and data analytics to create smart thermal treatment systems.

Considering the need for more efficient thermal systems and a better understanding of electricity generation in this regard, this review is presented. This revision has focused on pyrolysis [5], gasification, and combustion, in addition to the technologies used to control thermal treatments. In the review presented here, four main publishers were considered based on research on Google Academic: Elsevier, Springer, IEEE, and MDPI.

Thermochemical treatments are alternatives for municipal solid waste or biomass treatments, including combustion, pyrolysis, and gasification, which ensure the generation of three main products—electricity, fuels, and heat—with each treatment presenting advantages and disadvantages [6]. The main difference between these technologies is in the oxygen inlet concentration, which feeds the reactors, produces different thermal routes, and consequently changes the products, including into fuels and hazardous gaseous emissions, in the power plants [7].

All these alternatives need different residence times for the stability of the process, and this period can determine the different products generated [8]. In pyrolysis treatments, the reactor mainly depends on this variable, and thus it has considerable influence on the products. It can produce synthesis gas, bio-oil, and biochar with different concentrations in slow pyrolysis, fast pyrolysis, and flash pyrolysis reactors [9]. Synthesis gas, or syngas, is a fuel constituted mainly of carbon monoxide and hydrogen that generally can be used for electricity and/or heat production, has energetic potential transformations from chemical synthesis, such as through the Fischer–Tropsch process, and can become gasoline, methanol, kerosene, or other petrochemical derivates [10].

Energy recovery with syngas chemical looping is an important strategy for municipal solid waste or biomass in thermal or biological treatments. This solution consists of feeding gas from steam turbine cycles, which is a feasible alternative to biogas, providing anaerobic digestion or synthesis gas generated by thermal treatments, such as pyrolysis or gasification, which can produce fuels in a biorefinery or generate electricity [11].

Biological treatments are widely studied, in particular, the use of anaerobic digestion in biogas production, organic waste production in agriculture, and livestock farming, and this process contributes to carbon dioxide (CO_2_) emission abatement and electricity generation [12]. It is evaluated that it could generate 4.5 to 6.9 GWh and avoid the emission of approximately 19.8 MtCO_2_/year. This treatment generates a waste called digestate.

Digestate is the matter that is not degraded in the process, and it generally constitutes 45% of the material inserted in the biodigester, a piece of equipment that contains the microorganisms responsible for the anaerobic digestion process [13]. Pyrolysis is a thermal treatment that has been suggested for the generation of electricity with digestate due to the sustainability of this thermal treatment and low gaseous emissions. It has been estimated that the symbiosis of pyrolysis and anaerobic digestion could increase the amount of electricity generated by 42% compared to a stand-alone microorganism process [14].

Pyrolysis is a sustainable process that is applied to organic waste until dangerous chemical waste generates electricity and forms biochar, which is a byproduct that is used in soil conditioning and carbon capture systems [15]. Incineration with energy recovery in a steam cycle is the most popular technology worldwide. In small and medium plants, it is interesting to apply cogeneration to provide economic savings [16]. However, generally, this presents hazardous environmental gaseous emissions, such as nitrogen oxides and sulfur oxides (SOx), according to the waste inserted, and a lower efficiency is estimated at less than 70% in combined heat and power concerning gasification and toxic metal concentration in the ash generation process [17].

Slow pyrolysis is a process with excellent thermodynamic efficiency and is used for solid waste, biomass, and heterogeneous materials with different heating values, granulometry, and dimensions that generally reduce the power overall, as in gasification systems [18]. For this, this technology produces syngas in gasification, which reduces the disturbance to the power system and lowers the environmental impact compared to other time-residence pyrolysis treatments, allowing the insertion of biomass and solid waste without limitations or prejudice [19].

Astrup et al. [20] identified different research studies compared in a life cycle assessment of different thermal waste technologies, and noted the information gap on gas generation depending on the solid waste type in all the main thermal treatments: incineration, gasification, and pyrolysis. Biomass and/or solid waste power plants are extremely sensitive to the quality of fuel according to moisture content and composition, in particular in thermal reactors. Due to these particularities, a dynamic analysis and simulation of the electric machine become essential steps for attaining desirable performance in the electricity market [21].

**Figure 1:**
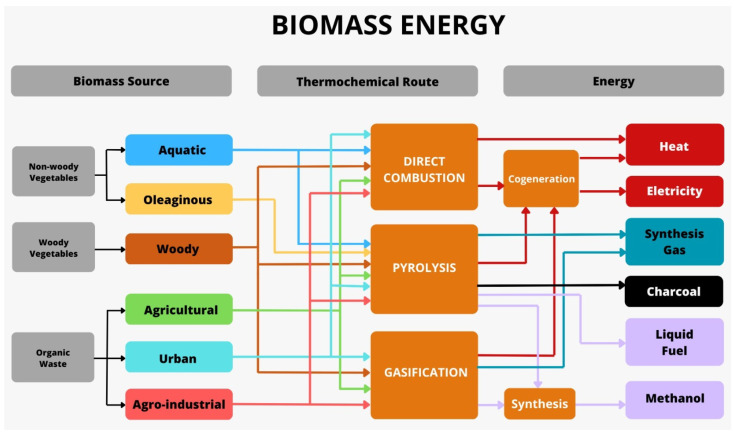
Flexibility of the biomass sources.

Fast load ramps characterize this need to enhance competitiveness in grid stability and to work together in the process modeling of feedstock materials [22]. Figure 1 shows the flexibility of the biomass sources that can be treated through so-called thermochemical routes (direct combustion, pyrolysis, and gasification), as well as the versatility of the energy obtained, ranging from liquid fuels (fuel oil and methanol), solid fuels (coal), gases (synthesis gas), heat, and electricity.

Thermochemical technologies use three sources of biomass: non-woody plants (aquatic and oil plants), woody plants (wood), and organic waste or waste with hydrocarbons in its composition (agricultural, urban, and agro-industrial). When it comes to municipal solid waste, it is worth noting that the residual plastic fraction is also part of this composition, as it is made up of hydrocarbons [23].

Pre-treatments have been developed and present possibilities to homogenize important parameters in biomass that increase the energy and electricity potential. These are divided into physical, thermal, chemical, and biological, and allow equalized moisture, particle size, lignin, and mineral matter content to contribute to the production of an equivalent and linear syngas yield of electricity generation [24].

Research about syngas is included in the current literature. The biomass potential to generate electricity with syngas in Brazil in the Rio Grande do Sul with Pelotas’ rice industries represents a capacity of 7.7 MWh by using the rice husk and effluents and could make these industries self-sufficient [25].

The energy balances of biomass or solid waste thermal treatment by slow pyrolysis plants in rotatory kilns have been applied in modeling works that study the process behavior of biomass and solid waste treatment [26]. It evaluated that slow pyrolysis works with low-grade, pretreated biomass and becomes self-sufficient after the insertion of biomass [27].

A few variables are evaluated in a single thermochemical technology and are controlled by operators since there are more than 100 process parameters to improve reliability and conditions to overcome the main challenges, which are recovering energy losses and understanding the possibilities during the energy conversion to increase efficiency as well as profitability [28].

In slow pyrolysis power plants, there are many different applications due to syngas versatility, such as the potential to generate steam, biofuels, and electricity, and these generally conflict simultaneously in daily and monthly price markets and in the necessity of supply chain-associated design and operation to identify the most cost-effective and sustainable reaction pathways [29].

Due to the versatility of the produced fuel (syngas), it is interesting to determine which application presents more advantages for a specific industry depending on the objectives and the supply chain by applying multi-objective optimization to make the waste treatment process rentable and feasible for different electrical systems [30].

Carbonaceous materials of biomass treated by thermal processes, such as pyrolysis, can be converted and have the potential to manufacture nano-materials for gas sensors due to their chemical flexibility and good electrical conductivity. However, a flexible research approach is needed utilizing portable detectors to identify the advantages of their possible long-term re-usability, which is beneficial when using biomass waste nowadays as it offers a more feasible approach to the market [31].

The production of this carbonaceous material in combination with hydrogen storage capacities and chemical combination with metal hydrides is a future strategy for the export of this material and can consequently contribute to electricity generation from hydrogen to power in the country of manufacturing as well [32].

The remainder of this paper is organized as follows: In Section 2, Section 3 and Section 4, pyrolysis, gasification, and combustion are explained, respectively. These are the main forms of thermal electricity generation currently used and are therefore covered here. In Section 5, the most important characteristics of thermal energy generation are presented and explained. In Section 6, the most important concepts for the control and automation of power plants are discussed. In Section 7, final considerations and possible future work are presented.

## 2. Pyrolysis

**Figure 2:**
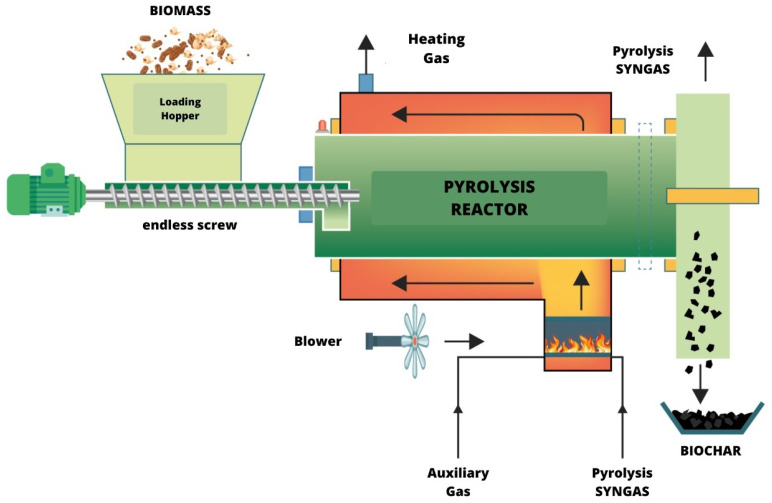
Cyclical process of pyrolysis.

Thermal decomposition in the treatment of sewage sludge in pyrolysis reactions does not present oxygen content inside the reactor, and due to this, the level of carbon dioxide emissions is reduced in addition to presenting economically marketable products, such as synthesis gas, bio-oil, and biochar, whose percentage composition is dependent on the chosen pyrolysis route [33]. The cycle of pyrolysis is presented in Figure 2.

The reaction time and heating rate are the main parameters that intrinsically divide the pyrolysis process into slow or fast, modifying the yield and the products generated when it comes to the two most commonly used technological routes [34]. However, the capture of carbon dioxide in either of these two routes with the addition of different catalysts and the influence of these parameters being researched is unknown because it is a recently emerging topic. This is amplified by the difficulty of recovering the chemical compound applied as a catalyst and of its mechanisms differing according to its allocation in situ or ex-situ. From this second mode, it is recovered more easily since the catalyst does not mix inside the main pyrolysis reactor, promoting greater filtration [35].

A reaction that has two raw materials inserted in the reactor is called co-pyrolysis. If organic products and water form, after reforming the steam, they produce free hydrogen as gaseous fuel, which mainly occurs in slow pyrolysis [36]. Reducing the rate of carbon and oxygen in the biomass depends on the reaction time, which affects the properties and yields of the products generated in the pyrolysis process [37]. The performance and interferences in the capture of carbon in the various technologies that are being researched are unknown and have recently been applied at industrial and experimental levels [38].

Small electricity-generating units are more commonly applied to the thermochemical routes of pyrolysis and gasification, which allow the compaction of plants by techniques such as incineration, thereby facilitating their installation close to the source of the biomass or raw material [39]. This material is then oxidized and can be reused in the form of the heat generated or as by-products, such as biochar or bio-oil, in various energy applications and technological products, such as gas adsorbents, fuel cells, activated carbon, and carbon sequestrated by the soil [40].

In the pyrolysis process, there are three main parameters evaluated in the percentage composition of products generated, which are the heating rate, temperature, and residence time [41]. They are interdependent on the chemical and physical reactions of this complex process and are also involved in the geometry of the reactor and the system of supply [42]. Secondary factors such as particle size and pressure are evaluated to avoid corrosion of the equipment that reduces its useful life period [43].

Pyrolysis reactions via microwaves are carried out by controlling the temperature or dielectric power, which starts with temperatures of 200 °C. However, it can reach heating ranges of up to 800 °C or electrical power of 1200 Watts. Analysis in real-time of the temperature increases efficiency in biofuel production, and the evaporation rate is a crucial parameter in the mechanism of mass transfer and absorption of consumed energy [44]. Applying catalysts that increase the heating rate and the cracking of larger molecules contributed to the yield of the process [45]. The application of alkaline catalysts such as sodium hydroxide in a hydrothermal gasification reactor at temperatures of 632 to 717 °C improves the performance and yield of fuel gas production, and the conjugation of algae can perform sequential fixation and sequestration of pollutant carbon to increase energy recovery [46].

The purification of the gas or sludge inserted must be carried out if there is a concentration of heavy metals, since biological organisms cannot withstand these adverse conditions. When catalysis by mineralization is carried out only in the improvement of the biochar of the pyrolysis process, it is necessary to evaluate the associated costs in the current currency in the acquisition of carbonates, silicates, phosphates, and hydroxides and of this doping in the removal of carbon dioxide by the soil in CO_2_/ton [47].

## 3. Gasification

The method of gasifying renewable inputs to produce fossil fuels by chemical processes with low levels of oxidation inside different models of reactor construction presents a series of modes of operation described in the literature [48]. Verification of the application of technologies’ concomitant processes that aim at higher yields and lower pollutant emissions is being widely researched through the connection of biological routes [47].

**Figure 3:**
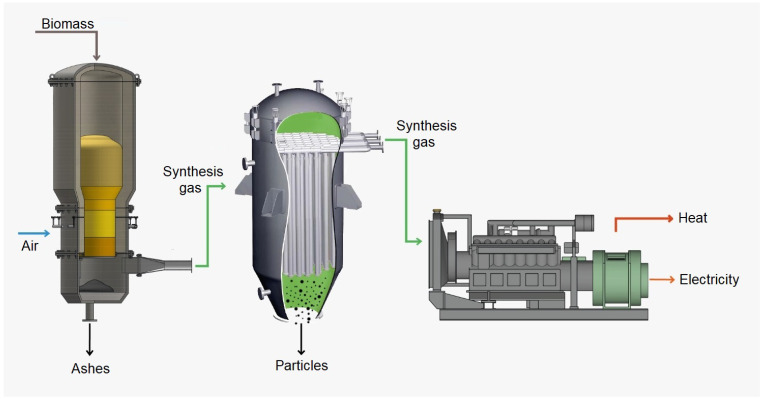
Cyclical process of gasification.

Microalgae has been applied to decarbonization in gasification technology and waste heat recovery in conjunction with the cooling of synthesis gas. The fuel generated in the process preceding the mixture in the engine transforms mechanical energy into electrical energy, enabling thermal efficiency, electricity generation, and a reduction in pollutants [49]. The cyclical process of gasification for power generation is presented in Figure 3.

In addition to the joint use of biological processes, one can resort to reducing carbon dioxide emissions within the gasification of biomass through chemical activities such as capture by compounds produced at an industrial level, such as calcium carbonate and non-amine solvents [50].

Solubilize part of a concentration of polluting gases at the same time as performing computational simulations and mathematical modeling on the process, which together can detect similarities. After validation with experimental tests through the collaboration of estimates based mainly on energy levels and/or the conservation of mass and gaseous compounds generated in thermal reactions, this process can be applied to reduce medium-term costs and contribute to the automation of the reactor [51].

Some residues, such as tar, ash, and particulates, are generated in sludge gasification and are disposed of in landfills. The toxicity of these residues requires higher classes of landfills for disposal, demanding a higher monthly operating cost of post-thermal treatments, which can make the process unfeasible, impairing the environmental and electrical efficiency achieved with the energy use of the sludge, which contributes to sustainability and the circular economy [52].

In this way, research on the application of these residues promotes their economic viability by making them into by-products after the application of chemical or biological treatments, such as adsorbent material or phosphorus recovery, among other alternatives, which will depend on the degree of investment required, plant size, and recovery period through the evaluation and demand of consumer markets [53].

Carbon capture and storage (CCS) in incineration technology presents the most industrially accepted degree of commercial evolution [54], considering that there are around 1200 plants in operation with high economic viability, mainly in large-scale projects. Incineration reduces the efficiency of electrical generation when using cryogenic distillation, chemical air separation, or recirculation of fuel gases [55].

When applying the most usual processes on an industrial level, the verification and evolution of studies that present new technologies can contribute to possible gains in other characteristics of the power plants currently in operation [56]. These studies mainly evaluate the integration of systems and visualization tools in real-time, apply sensors with process control in machine learning, and research the use of advanced materials and catalysts [57].

In European countries like Austria, Slovenia, Germany, Greece, Belgium, and the Netherlands, incineration plants are nowadays the most commonly used method of thermal treatment of municipal sewage sludge, in conjunction with co-incineration in power plants that use coal as the raw material in cement kilns [58]. Technologies such as gasification and pyrolysis emerge as future possibilities, mainly on smaller scales. In Portugal, the use of heat in any of the aforementioned technological options in a centralized way and at strategic points increases the electrical power output [59]. This automatically contributes to the maintenance of operations for long periods since they have greater geographic, social, economic, and environmental functions [60].

Checking efficiency rates and finding chemical compounds that can dilute the concentration of carbon dioxide emitted and its conversion by mass transfer into a commercial or easy-to-treat by-product can reduce environmental pollution at its final disposal [61]. This application depends on the evaluation of the concept in on-site production, reuse, and storage, the economic perspective of operating costs, and investments in the creation of new technologies or the application of commercial routes, such as separation by catalytic and non-catalytic selective reduction and dry adsorption. It must be combined with the treatment of other pollutants generated in incineration, such as fly ash [62].

The conception of new technological possibilities is important because innovation aims to reduce existing bottlenecks and promote greater environmental sustainability [63]. With cost reduction and greater operational efficiency in thermal treatments of municipal or industrial sewage sludge [44], recent alternatives include the supercritical oxidation of water, microwave-assisted pyrolysis, and plasma pyro-gasification [64].

Within the equipment that performs sludge oxidation in high-pressure atmospheres, generally reaching 22 to 25 MPa and average temperatures around 400 °C, there are several challenges to becoming a commercially viable technology, including its high investment costs, operating time, and oxygen consumption compared to other recent technological alternatives [65]. As an old equipment type, there are constant incidences of corrosion and factors associated with the intensity of the process. There are precautions for the viscosity of the sludge by transporting it in pipes and at high humidity, which increases the production of hydrogen and reduces methane; however, this also decreases the calorific value of the gaseous products generated [63].

Plasma has different technological aspects and can perform the treatment of organic and inert material when it is combined with gasification and pyrolysis in a plasma torch, which reaches internal temperatures of 1500 °C. It can recover dangerous materials, such as cadmium, lead, zinc, and chromium, which may exist in some types of sludge, such as tanneries, in which acidic reagents are applied; among them are calcium and potassium oxides with water, which are filtered in a microfiltration system [39]. The recovery of these chemicals allows reuse and an evaluation of the reduction in costs and environmental impact. However, this technology has limitations arising from the erosion of electrodes by some fuels, and it requires high energy consumption to start the operation despite its versatile treatment capacity with hazardous waste [64].

In thermochemical routes, when waste is inserted as raw material, the average energy efficiency achieved among the highlighted technologies is 30%. In addition to electricity, other products with varied economic value, such as fuels, renewable gases, and chemicals, can be derived from the use of catalysts [66]. Therefore, these processes can expand the global yield rate and possibilities for greater gains in scale in the long term according to the demand for each product generated in the plant and its need in the national or global market. In this context, automation can favor operational constancy when the best efficiency range is found, reducing disturbances and losses due to inertia [67].

The selection of the reactor depending on the specifications of the inserted raw material and desired products is a factor that determines the success of the thermochemical transformation process and the automation [55]. To continuously reproduce the same results in a way that reaches the degree of desired or required energy efficiency depends on the improvement of the machine design according to experiments and multiphase simulations. To guarantee and improve longevity with advanced materials, loss monitoring by sensors and automatic control are integrated into process optimization [68].

After the selection of the technology and reactor types, the characterization of samples and control diagnosis are carried out in each of the three categories of the capture and separation of carbon dioxide produced in the thermal process, which are divided into pre-filtering combustion, post-combustion, and with oxygen or fuel and catalysts [69]. When evaluating the best relationship, automatic control permits the reduction of operator training, maximizing economic benefits, and improving process safety as it provides the greatest guarantee of reducing pollutants generated in the system [70].

In the context of automation applications, several models depend on the desired degree of control. There are several models that can cause variability and conjunctions, with more than one algorithm applied according to the strategy, which can be adaptive, predictive, intelligent, and automatically coordinated, and use logic and mathematics to reach the desired operational conditions [53].

Being dependent on the investments that can be made, the requirements necessary and the possible manipulation of operating variables—since the process settings are according to capacity, size, type of material to be processed, and products that aim to be generated—are crucial to the favorable financial viability of the project [69].

Sensor data in a control interface with real-time graphs and historical data provide reports that can provide information that assists in the operation of drive logic, diagnosing and predicting possible failures [71]. They can avoid and minimize the possibility of sudden stops and adjust the air supply by limiting the formation of atmospheric, water, and soil pollutants [72].

The control interface for these systems can be based on human–machine interfaces (HMIs), graphical user interfaces (GUIs), monitoring systems, or supervisory control and data acquisition (SCADA) systems. These interfaces have the goal of presenting or receiving information in a friendly way for the user. This facilitates the updates, measurements, and controls needed in industrial systems [73].

Platforms that carry out data analysis and processing generate expected improvements in the performance of thermal processes through the increase in the calorific value of the synthesis gas, which increases the generation of electricity, and in the temperature of the flue gas, which affects the reduction of pollutants [74].

The calibration of sensors provides measurement and control through the creation of alarms and the detection of optimal ranges. They can benefit from mechanisms for capturing and filtering the volume of harmful and non-combustible gases with control loops that regulate the concentration of sludge and cool the generated gas. This could provide automatic adjustments in the feeding system, especially if there is a recurrent use of centrifugal pumps in this operation [75].

The growth in the scale of renewable energy production with sustainable gas generation by waste can provide an efficient solution for reducing costs and benefits to society related to energy demand by acting on the supply problem, being able to guarantee safety in electrification, and reducing the transport of toxic or non-toxic sludge in remote locations such as landfills that are generally distant due to the need for ample disposal space [76].

When automation was applied after carrying out several tests and having an efficient and financially viable control diagnosis, it allowed the expansion of different operation sizes and the staging of the operation of plants more easily and quickly with a lower level of technical instruction than in a non-automated and manually operated system. This avoids the greater probability of human errors and provides greater operational safety and electricity generation with materials that are currently discarded as waste in the soil [77].

Mechanisms for purifying and cooling the gases mixed in the engine constitute an important step in the previous improvement of electronic and controlled fuel injection since volumetric efficiency and less-mixed loads benefit the design of rotating thermal machines and the simulation of fuel models [78].

## 4. Combustion

**Figure 4:**
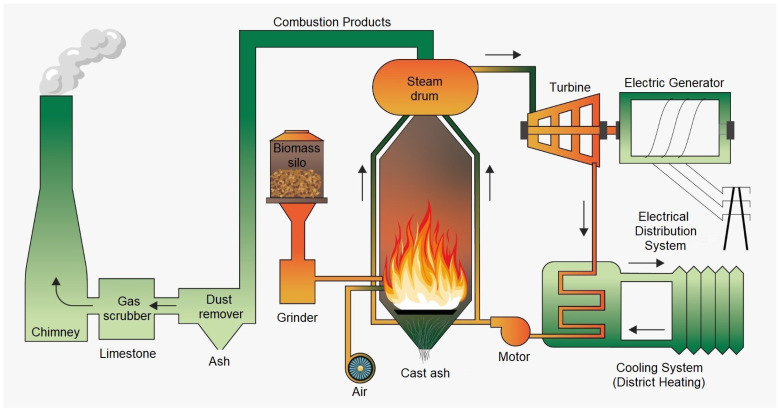
Cyclical process of combustion.

In combustion, the reduction in the mechanical use of parts with the automation of the flow is allied to the interconnection of the electrical power generated (from sensors), the temperature of the exit gas, and the volumetric concentration of each gas. These sensors transmit this information, and that will allow us to prove the adjustments’ relationship between electrical generation × gas cooling × gas separation in the thermal process, applied mainly when aiming at the chemical fraction of hydrogen [79]. The process cycle of combustion for thermal generation is presented in Figure 4.

The performance of installations that have carbon capture depends on the evaluation of the release of chemical energy during combustion and the influence on the volume and location of oxygen [46]. According to the particular design of the combustors and the heat transfer measured, by applying statistical analysis as a tool in assisting automation, it is possible to diagnose the control strategies with the highest yield, lowest oscillation, and maximum overshoot values automatically assigned in the operation [80] through the relationship between the automated insertion and the cost of the minerals applied in the removal of carbon dioxide [51].

It is possible to minimize the production of sludge and the challenges of providing treatment and ensuring water quality by combining the capture, storage, and transformation of atmospheric pollutants with disposal to generate a lower environmental impact. This can occur through different mechanisms, such as the use of chemical catalysts, drying, and advances in primary and secondary treatment technology in biological reactors [81]. In addition, there are techniques that promote the reduction of transport costs, which is a factor that reduces the emission of pollutants, such as by thickening, that is, becoming denser and reducing the space used in vehicles that carry out the disposal or treatment of the sludge load [82].

When moisture reduction is carried out by drying mechanical or thermal drives, the CCS improves. This decision depends on the demand for power and the correlation between the possible energy gains and the necessary investment, in addition to the fact that, in sludge production, one must verify the financial economy in the disposition of this matter, as all these aspects follow the objectives of the world climate [83].

Having evaluated the impact of the automatic control on the quality of the dehumidifying process, it is needed to act on the speed of rotation of the drying in the engine with the most efficient frequency conversion on the compound in which it is wanted to improve the biological or thermal treatment [84]. After the reduction of the water content, the release of nutrients is consequently increased, and the content of heavy metals is reduced before the execution of the treatment, which, through experimental tests, can evaluate the level of improvement achieved in these two parameters [85].

Due to the reduced water content in the sewage sludge, the energy spent on transporting the material decreases, as it provides greater logistical space in terms of both disposal and treatment. In a unit that uses part of the residual heat in this drying or a heat supply with a biological treatment, such as anaerobic biodigestors, or a thermal treatment, such as pyrolysis, gasification, or incineration, carbon dioxide emissions were minimized in values above 50% by evaluating all the steps mentioned [76].

An improvement in this performance should be verified regarding the economic and investment advantages and disadvantages of applying methods such as thickening, mechanical dehumidification, and conditioning that enable a moisture content in the range of 70 to 90% while thermal drying. These can guarantee water content between 5 and 10% and the generation of products in each treatment of the inserted sludge [86].

Applying catalysts after drying the sludge depends on the selection of reactors and on the verification of the performance of each adsorbent in its chemical transformation through gas–solid contact, which can be fixed, fluidized, mobile, or rotating in the reactor, in addition to evaluating the minimization of capital and operational costs with the insertion of the chosen catalyst(s). Although increasing costs, this increases the generation of economic value and products refined by reforms and/or with load flexibility by a greater percentage, which makes effective the research efforts applied at an industrial level. In a way, this promotes the reduction of pollutant gas emissions after oxidation of the material and with a possible mixture of one or more combined catalysts [78].

When this occurs in a stable moisture content ratio, that is, with this constant variable, it collaborates in the automation of the advancement in rotational speed and the performance of brakes that are assigned to possible stops. It is proportional to the highest combustion and frequency of electric current generated with the drier sewage sludge. Thus, the fixed percentages of moisture lead to greater predictability in the control of these internal combustion engines adapted to synthesis gas and coupled to electric generators by different catalysts, both through subdividing and statistically. Verification of the emission reduction potential of polluting gases is performed by parameterizing the applied catalyst/sludge moisture, pressure, initial and average temperature, along with residence time [87].

Absorption and desorption columns constitute important structures within the existing mechanisms of carbon capture. These must be conditioned and their volumes calculated regarding the necessary periodic charge of chemical compounds or solvents that will be applied on an industrial or experimental scale. Storage facilities were observed according to the level of danger, market demand research, and programmatic operational needs [88], together with the load flexibility of the electric-power-generating plants and the energy that will be commercialized in front of the automation of the data and the machine that collaborates in the design of the strategies that will be applied [89].

## 5. Important Features for Thermal Power Generation

Thermal power generation involves the conversion of heat energy into electrical power and relies on various features for efficient operation: **Heat source:** a consistent and reliable heat source, such as fossil fuels (coal, natural gas) or renewable sources, like geothermal or solar energy, is fundamental; **cooling systems:** effective cooling mechanisms prevent overheating and maintain operational efficiency, often using water or air-cooling methods; **control systems:** precise monitoring and control systems regulate temperature, pressure, and other critical parameters to ensure safe and optimal operation [90].

### 5.1. Temperature Influence on Thermal Generation

In two-stage pyrolysis reactors, the elevation of temperatures contributes to the increase in the thermal cracking of solids and liquids, facilitating their subsequent use, with the hydrogen and carbon monoxide contents and the dry gas yield increasing up to the 850 °C range at temperatures starting at 500 °C, and the use of catalysts such as calcined dolomite can reduce the tar content and produce a higher concentration of synthesis gas in the catalytic conversion of carbon from the inserted biomasses [91].

Conditions of up to 550 °C, through modeling at a heating rate of 15 °C/min, present the tendency to increase energy recovery by biofuel, both bio-oil and biochar, from sewage sludge with moisture contents up to 15% in conventional pyrolysis processes [92]. In addition to the combustible gases produced, which are one of the main products in the pyrolysis route and are directly influenced by temperature, the properties of biochar derived from sewage sludge include that the elevation of this parameter reduces the production of this compound, that is, lower temperatures around 300 to 400 °C increase the yield or concentration of biochar in the percentage of economic products generated in the pyrolysis process [93].

The functions of this material vary, and biochar produced at low temperatures can be viable in the correction of alkaline soils, while those generated at higher temperatures (700 °C) can be more active in improving soil fertility and carbon sequestration by reducing the necessary volume of fertilizers. By interconnecting incineration or combustion technology with pyrolysis, biochar can be an oxidizer that can produce and provide heat in the drying of sludge, and the ash generated can be marketed as phosphorus fertilizer [94].

### 5.2. Constant Pressure Importance

It is verified that, in the processes of pyrolysis and gasification, keeping the pressure constant or linear also guarantees the stabilization of the composition of the products generated, mainly gases as well as residues, according to the average molecular weight [95]. This contributes to the ease of condensation and the cooling of the gas generated, which has favorable combustion in internal combustion engines that transform the mechanical rotation into electrical energy with greater load constancies, partially expending this energy load in heat [96].

Depending on the coating material, part of this thermal energy can be stored and be used in the preheating of the engine. Pressures above 2 to 3 MPa produce free hydrogen in pyrolysis when using biomass, reducing the oxygen and carbon ratio in the vegetable bio-oil produced and increasing the combustion potential. Several important aspects improve energy systems, especially regarding smart grids [97].

The pressure drop inside the reactors modifies the permeability of the gas and also provides for the need for adjustments in the control of the residence time of the inserted material and the balancing with the temperature and the electrical energy that must be delivered to the network according to the contracted sale and that will be marketed. Pressure in the range of 3 to 7 MPa is recommended in the pyrolysis process to enable greater thermal decomposition of the tar and to raise the yield of the synthesis gas of the municipal sewage sludge [98].

It is verified that control and automation become crucial in these points of demand, avoiding social and economic wear and tear of the thermal operations of the pressure exerted in the reactor, together with the analysis of factors and predictability of risks and alarms already previously configured in worrying unit values of pressure, which allow a reduction in the possibility of the occurrence of various operational problems [98].

### 5.3. Heating Rate on Thermal Generation

When a heating rate of 10 °C/min is adhered to in the pyrolysis process on a laboratory scale and carbon dioxide is used as a reactive medium gas, the concentration of carbon monoxide is raised at temperatures above 550 °C, and less tar is generated because the cracking of volatile compounds increases. Although, when it is desired to increase the total yield of gaseous products and bio-oil on industrial scales, higher heating rates are generally applied, as previously mentioned, in the range of 15 °C to 30 °C/min, which is justified because it is a sudden and high variation reaching the maximum and optimal temperatures of 500 °C to 800 °C, which also contributes to increasing the energy density of the synthesis gas in the conventional and slow pyrolysis technology models [99].

These heating rates of 10 °C to 30 °C are low compared to fast pyrolysis or flash technology, in which the residence time is only seconds and heating values exceeding 100 °C per second are achieved. In this route, a greater volume of bio-oil is produced concerning synthesis gas and biochar. Likewise, when evaluating gasification on laboratory scales, smaller-scale heating, such as from 5 °C to 15 °C/min, is applied to provide the behavior of the operation with a higher degree of analysis in different temperature ranges by statistically employing thermogravimetric analyzers. While in the operation of fixed or fluidized commercial gasifiers, they reach temperatures above 1000 °C in complete tests in times of less than 20 min [100].

The drying of the sludge depending on the mechanical equipment used provides different particle sizes in millimeters that are distributed in the dryer and collected according to its capacity in tons per day, energy consumption of heat and electricity, and ability to generate particles from 0.02 mm to 10 mm. Among these can be highlighted the spray dryer, which generates compounds of smaller granulometry, and the paddle dryer, which generates larger particles [84].

The prior choice of the sludge particle size and designated equipment is essential because it must be integrated into the feeding system of the thermal treatment reactor, although this choice criterion must be evaluated together with an analysis of the chemical and physical compositions of the sludge since these conditions affect the optimal ranges of heat distribution in the thermal treatment that will be performed [62].

The evaluation of the instantaneous feeding rate in thermal processes, such as gasification and combustion, is a vital task that monitors pelletized fuels from a vision machine in measurement time, showing density and diameter influence applied to other parameters working together with the oxidant feed rate to establish improvement in control and their relation with the gaseous and carbonaceous products generated [101].

Torrefaction consists of another technique widely used in the pretreatment of sludge. It is mainly applied when particles up to 1 mm are obtained, which reduces the emission of pollutants such as SOx in the pyrolysis and gasification processes because, when the elemental analysis is performed and compares torrified sludge with conventional sludge [102], the concentrations of sulfur are reduced due to the devolatilization provided [103].

The energy density is high according to the increase in the upper calorific value of the generated compound and drastically increases the concentration of hydrogen, methane, and carbon monoxide generated. In addition, this material obtains better fertilizer characteristics by releasing higher concentrations of carbon, magnesium, and potassium in its pores [104].

Considered one of the most relevant factors in thermal processes and which subdivides the technological routes when integrated with the programmed temperature to be reached, that is, the maximum temperature of the process, also called reaction temperature, the residence time of the raw material becomes the preponderant characteristic of the final composition of the products that will be generated and marketed [42].

### 5.4. Time Importance in Thermal Power Plants

Thermal power plants have specific start-up and shutdown procedures that take time. Start-up times can affect the responsiveness of the plant to sudden increases in demand, while shutdown times are important for maintenance and other operational considerations. Managing these times efficiently is crucial for maintaining a stable and reliable power supply [105]. Scheduled maintenance of thermal power plants is essential for ensuring optimal performance and preventing breakdowns. Time-based maintenance schedules are designed to address wear and tear, replace components, and ensure the overall reliability of the plant. Planning these maintenance activities during periods of lower demand helps minimize disruptions to the power supply [106].

Because it is closely linked to the heating rate, precautions that must be taken with the final temperature that will be adopted also impact the composition of elements, such as potassium, magnesium, iron, phosphorus, and silicon, in the ash or biochar generated. Although, in technologies such as slow pyrolysis that compare the temperature and heating rate with the residence time, this aspect is considered of greater relevance only when low temperatures around 300 °C are applied in about 20 min of residence and loses importance when the reaction temperature reaches values above 400 °C when referring mainly to the generated biochar [107].

The authors of [108] evaluated a negative carbon dioxide power plant through a gasification unit in the mathematical modeling of reactions with sewage sludge by integrating three different software types—Aspen Plus (responsible for thermodynamic simulation), Excel, and Aspen Hysys (model and energy balance)—and validated that temperatures from 600 °C to 760 °C directly influence the pressure of the system, being that the highest simulated temperature provided a greater concentration and formation of combustible gas in the reactor, which is carbon monoxide, than of carbon dioxide. They reported in their conclusions that temperature is the most relevant factor following the gasifying agent (water in the form of steam) and pressure.

## 6. Power Plant Control and Automation

In a simulation with electrical generation in a computerized gasification model, two main conditions are determined: the ideal equivalence ratio and the optimum reaction temperature of the process. From these, the relationship between the gasification agent that allows oxidation, which can be air, oxygen, or steam, and the volume of fuel or raw material inserted validates the main temperatures that promote the superior calorific value of the synthesis gas generated and the thermal power available to be generated as a function of the energy required to preheat the gasification agent [109].

One of the possibilities of calculating the equivalence ratio is by PLC instructions, which, from the responses of the anemometer that measures the air velocity, define the designated flow rate in a vacuum pump that will feed the raw material into the gasifier through an inverter and a proportional–integral–derivative function. The average electrical efficiency of gasification is around 29% in conventional diesel oil, while the gasifier yield approaches 45% with the use of air in the gasification of sewage sludge. This 16% reduction occurs due to heat losses and mechanical wear, but it can be mitigated with the automatic control of the ideal equivalence ratio that raises the lower calorific value of the synthesis gas [110].

PLCs play a crucial role in the control and automation of thermal-electricity-generation systems. These systems typically involve the use of boilers, turbines, generators, and other components to convert thermal energy into electrical power. PLCs are employed to monitor, control, and optimize the various processes within the power plant. Using PLCs in thermal electricity generation provides flexibility, reliability, and efficiency in managing complex processes, contributing to the overall performance and safety of the power plant [111].

In control programming, advanced linear and nonlinear strategies can be adopted in the performance that an electric generation unit will have in its behavior concerning CCS mechanisms. And, depending on the cycle and stage of the process, it can activate the actuators designated in a predictive mode of the table of variables considered in the application and in the number of steps established that is dependent on the evaluation of the control horizon and forecast as the required computational demand [112].

Industrial communication networks enable devices, machines, and systems to communicate and share data, facilitating the automation, monitoring, and control of industrial processes. Several communication protocols are used in industrial settings to ensure reliable and efficient data exchange, such as transmission control protocol/internet protocol (TCP/IP), Modbus, and object linking and embedding for process control (OPC). TCP/IP serves as the foundational communication protocol for networking in industrial environments, Modbus is a specific communication protocol used for connecting industrial devices, and OPC provides a standardized framework for communication and data exchange between different components in industrial automation [113].

In this context, the IoT has been applied for network automation [114].

IoT applications and their power to increase efficiency across multiple industries by leveraging real-time data have allowed centralized control and management of various devices, optimizing the energy consumption of power plants, enhancing supply chain productivity, and reducing downtime with predictive maintenance [115].

In this way, these samples must be reviewed continuously to verify the stability of the associated electrical demand frequency, mainly to reduce sudden oscillations that can generate fines and greater atmospheric and environmental pollution [116,117,118]. By performing several tests, a robust database can be created among different operating modes and repeat calibration in engine speed, load, architecture, and control and lower braking potential, which consequently reduces nitric oxide and nitrogen dioxide emissions and promotes improvement in electrical power output [119,120,121].

Supervisory control equipment is vital, especially in non-linear systems that have several measurable variables and sudden changes, as it assists in the visual control of the operation and improved detection of alarms of risks and optimal ranges desirable by languages such as functional block diagrams and structured text [122].

Given that electricity generation involves industrial systems, automation and communication concepts are applied [123]. As presented in [124], IoT systems can be used with success in energy management, proving how connected these technologies are. Observing that Industry 4.0 is more present in these applications, the programmable logical controller (PLC) needs to be used for these solutions, where IoT-PLC can be an optimal solution [125]. In addition to the traditional thermal systems, hydrogen generators are a possible way to produce energy [126].

Gas sensors in thermal processes are available at temperatures up to 1000 °C, collaborating with K-type thermocouples inside the reactors, as in pyrolysis or gasification processes, which is important to determine some gases according to temperature with good stability over a long time and to selectivity detect methane, hydrogen, and carbon monoxide. However, the highest temperature remains a problem for their lifetimes, which needs to be solved by researchers and which is contoured with modulated gas multi-sensor arrays where artificial intelligence can be used to reduce noise [127].

When developing an inspection application, the need for scalability in conjunction with cost-effective integration is highlighted for accuracy, reliability, interoperability, and security in the reproduction and transmission of data. The insertion of the programmable logic controller allows for the production and management of several routes and automatic methodologies according to the synchronization of information and creation of indexes with identification and weight calculations of the measured and control factors [128].

This is to be carried out periodically, aiming at medium-term savings in the management of the process by avoiding sudden stops in the operation of variable electrical loads in electricity-generating thermal units with the automated and most stable restart possible, thus providing benefits to the environment in combination with confidence in electricity commercialization [129].

### Artificial Intelligence Applications

When there is a nonlinear problem that needs to be optimized, a technique or algorithm can be applied in which continuous improvement and programmatic training occur, such as artificial neural networks [130]. With the modeling of the data received from sensors, performing equations relating to the thermal balance conjugated to the electrical balance through simulations and integrating them to diagnose losses and obtain the best basis of possibilities for adjustments in the system constitutes an important tool for reducing costs in an electricity generation system [131].

This allows us to verify which aspects of the raw material, thermal reactor, or electric generator have greater relevance in the electric generation that will be marketed as distributed and can guarantee greater global energy efficiency by adjusting the gain of the main factors that increase the electric power output. This is observed by [132], in which the authors verified, in a gasification system among 86 biomasses and 11 factors, that the temperatures in the gasifier and the elemental carbon analysis were the two main variables that corresponded to 25% of the electric power output among the 11 analyzed items.

The complexities associated with external factors influencing power production, like climate considerations, pose significant challenges in developing statistical models for managing generation systems [133]. Leveraging artificial intelligence (AI) techniques emerges as an enticing approach to addressing this issue. AI, a domain within computer science, demonstrates the capacity to comprehend tasks [134]; analyze data [135]; evaluate time series [136], optimization design [137], and classification tasks [138,139,140]; and make decisions using algorithms crafted by experts [141].

Furthermore, within this realm, extensive exploration has been undertaken in smart grids [142,143,144] and the realm of the IoT [145]. Within this framework, machine learning stands as a subset of AI focused on constructing algorithms through data learning rather than predefined instructions [146]. Ensemble learning methods amalgamate multiple machine learning models [147], employing techniques like averaging or weighted averages, to address the limitations associated with relying solely on a single model [148].

Reduced order models configured with machine learning as long and short-term memory networks (LSTM) are alternatives within an initial scope of obtaining autonomous plants because they are smaller programmed systems that can be added to chemical and environmental modeling from other software, forming a complex environment in which different programming languages are used. Among these, the Python language stands out for its results in bio-fuel minimum selling prices, evaluating mass and energy yields as necessary feed-stock volumes for commercial-scale power-plant planning [149]. As presented in [150] and in [151], the use of LSTM may be even enhanced by applying filters such as wavelet transform.

Support vector machines (SVMs) are AI modeling tools to evaluate hydrogen gas production from biomass pyrolysis for energy systems. They show that the highest temperature in the process is the variable that is most important in this machine learning technique. Compiling two algorithms together can increase the precision results: integrating SVMs with artificial bee colony optimizers obtained a better coefficient determination compared with M5 tree and multi-layer perceptron neural network algorithms [152].

Hybrid models with machine learning can play a smart operation role in gasification and pyrolysis, with possibilities of optimizing the operation, mainly due to catalytic material behavior, implementing rapid design, contributing to decision-making for multi-objective power plants, and increasing the yield of the desired output product [153].

The quest to develop biorefineries with autonomous control by advanced models based on the parameterization of embedded dynamic systems and with data servers that store, receive, and transmit operating data and that can apply minimization techniques and AI to generate acceptable emission levels and maximization techniques in the quality and quantity of fuel, fertilizers, electrical power, and chemical compounds generated is a global objective, especially in terms of achieving carbon neutrality: the effect of consuming the same amount of carbon produced during all stages of the process [154].

Advances in the training of machine learning algorithms that predict thermodynamic behavior, evaluate the immediate analysis of biomass by predicting the content of volatiles, ash, and fixed carbon, and automatically re-evaluate temperature, pressure, heating rates, and residence time, which consequently allow the desired approximate yield and the percentage obtained of each product, would help to adapt the marketing of commodities, and, with these measures previously calculated by AI models, would allow the economic modeling of the financial expenses and maximize profits of biorefineries from thermal power plants [155].

Predicting faults in electrical power systems improves their reliability, thus helping to increase customer satisfaction with the use of electricity. In [156], the wavelet transform was applied to improve the ability to predict faults in the electrical power system. The use of filters, such as in [157] and in [158] using seasonal trend decomposition, in [159] using wavelet transform, in [160] using Christiano-Fitzgerald random walk filter, and in [161] based on Hodrick–Prescott filter, is becoming popular since they reduce the noise and enhance the ability of the neural network to make predictions [136].

Applying the link between numerous linear and non-linear process factors is an important task to increase the accuracy of AI models [162]. Since the data amount depends on the quality of the calibration and hyper-parameter optimizations [163], the comparison difficulty concerning the performance varies according to the methodology and objectives used to reach data-driven models and can be improved with the advancement of deep analytical techniques and Internet of Things integration [164,165,166].

With the enhancement of computational power, AI models become capable of solving more difficult tasks [167,168,169]. Following this direction, there are more applications of sensors that are using AI approaches to improve their capacity of application, especially considering automation [170]. The use of these models in automation [171], along with sensors [172] and considering electrical power generation [173], will become even more popular given the direction of the trend of AI-based approaches [174].

## 7. Final Remarks

The comprehensive review of automation and sensors in controlling parameters for thermal treatments within electrical power generation underscores the transformative impact of technology on the power industry. Through the lens of this examination, it becomes evident that the integration of advanced automation and sensor systems plays a pivotal role in optimizing efficiency, ensuring safety, and enhancing sustainability in power generation processes.

This analysis has elucidated the vital role of automation in streamlining operations and reducing human error, thereby improving overall system reliability. Moreover, the critical function of sensors in monitoring and regulating essential parameters such as temperature, pressure, and flow rates cannot be overstated. These sensors enable real-time data acquisition, facilitating immediate adjustments and preventing system failures.

This review highlights the evolution of technological advancements, such as machine learning algorithms and IoT integration, which have revolutionized automation and sensor capabilities. These innovations have not only enhanced precision but also expanded the adaptability of control systems, leading to heightened performance and decreased environmental impact.

As electricity generation continues to face challenges of efficiency, sustainability, and reliability, the findings from this review emphasize the continued significance of investing in and further developing automation and sensor technologies. By leveraging these advancements, the power industry can continue its trajectory toward more efficient, safer, and environmentally conscious energy production for the future.

Since thermal generation is a solution when other renewable sources cannot be used considering their economic viability, more work needs to be conducted to improve the use of this energy source as cleanly and sustainably as possible. In future works, more evaluations regarding the use of cleaner ways of producing thermal energy would be promising to discuss this as an alternative when other power sources are not applied.

## Data Availability

Data are contained within the article.
